# Implementing a collaborative medicine and pharmacy educational activity in two countries

**DOI:** 10.1080/10872981.2020.1780697

**Published:** 2020-06-18

**Authors:** Tina Brock, Thao Vu, Amudha Kadirvelu, Chooi Yeng Lee, Fiona Kent

**Affiliations:** a Faculty of Pharmacy and Pharmaceutical Sciences, Monash University, Parkville, Australia; b Jeffrey Cheah School of Medicine and Health Sciences, Monash University Malaysia, Sunway, Malaysia; c School of Pharmacy, Monash University Malaysia, Sunway, Malaysia; d Faculty of Medicine, Nursing, and Health Sciences, Monash University, Clayton, Australia

**Keywords:** Education, medical, education, pharmacy, interprofessional relations, internationality, curriculum, instructional methods

## Abstract

**Background:**

To promote better collaboration for patient care, interprofessional education (IPE) is required in many health professions courses. However, successful IPE implementation at scale can be challenging because of complicated logistics and competing priorities. Implementing across multiple geographies adds further complexity.

**Objective:**

This paper describes the implementation of a full cohort IPE activity for medical and pharmacy students delivered at both the Australian and Malaysian campuses of Monash University.

**Design:**

We designed a 150-minute, blended learning activity centred around asthma care for second-year medical and pharmacy students. Student perceptions were measured with a pre- and post-activity survey using the validated ten-item, three-factor, SPICE-R2 instrument. Analysis focused on differences between professions and countries.

**Results:**

All second-year medicine (N = 301 in Australia and N = 107 in Malaysia) and pharmacy students (N = 168 in Australia and N = 117 in Malaysia) participated in the learning activity. A total of 326/693 (47%) students participated in the associated research by completing both the pre- and post-activity surveys. The pre-activity survey showed significant differences in four items between medicine and pharmacy students in Australia and two items in Malaysia. Post-activity, we observed significant changes in 8/10 items when the two professions were combined. Specifically, we noted changes across the countries in perceptions of roles and responsibilities for collaborative practice and patient outcomes from collaborative practice.

**Conclusions:**

IPE across different professions and countries is feasible. Positive outcomes in role understanding and perceived patient outcomes are achievable through a context-sensitive, locally driven approach to implementation. Longitudinal experiences may be required to influence perceptions of teamwork and team-based care.

## Introduction

To promote better collaboration for patient care, learning *about, from*, and *with* other members of the healthcare team is now an accreditation requirement for health professions training courses in many countries [1–6]. Despite this, the operationalisation of interprofessional education (IPE) initiatives varies widely[7]. This is, in part, because of logistical challenges and competing curricular priorities[8]. IPE activities involving multiple professions and large cohorts require careful planning and robust evaluation to justify their value and optimise their impact[9]. This can be even more complicated when designing for and teaching across different countries. In fact, despite globalisation of medical education [10–12], most interprofessional investigation has occurred in the context of a single country, most likely a developed country [7]. This paper presents insights from the first study of its type to implement a co-developed IPE activity based in a developed country, Australia, and a developing country, Malaysia.

This research specifically examines a large-scale instructional activity for second-year medical students and second-year pharmacy students collaborating on a multi-phase asthma-related case at the Australian and Malaysian campuses of Monash University. The structure of the programs at Monash University, where both the medicine and pharmacy courses have cohorts based in Australia and in Malaysia, provides the rare opportunity to develop, implement, and evaluate a similar IPE activity with two professions in these two countries. The research protocol was reviewed and approved (project number: 12166) by the Monash University Human Research Ethics Committee, inclusive of both campuses.

The design of the IPE activity in this research draws on a Collaborative Care Curriculum (CCC) framework which Monash University created in 2016 to scaffold the knowledge, skills, behaviours, and attitudes for the 12 health professions represented on its campuses[13]. The framework was developed with input from academics, practitioners, students, and consumer advocates. It encompasses four key learning themes – person-centred care, role understanding, interprofessional communication, and collaboration within and across teams. It sets learning outcomes for health professionals at the novice (first year), intermediate (middle years), and entry to practice (final years) levels of training. After launching the CCC framework, multiple new IPE activities (such as this one) have been aligned to the framework, to facilitate the educational design of developmentally appropriate content for all participating health professions. There was no intention to combine students from every health professional in every IPE activity.

The focus of this IPE activity in this research is the collaborative care for patients with asthma, which allows for authenticity and customisation, two important characteristics of successful IPE initiatives[14]. In identifying an authentic clinical context, we noted that the care for patients with asthma is suboptimal in many countries[15]. Prior research suggests the need for doctors and pharmacists to work together to improve long-term asthma control[16]. In both Australia and Malaysia, a key barrier to effective asthma counselling is a lack of role understanding among health professionals [17,18]. In Australia, the national asthma strategy promotes a holistic approach to the care of patients in order to reduce the impact of asthma on individuals, the community, and the economy[19]. This guidance suggests that initiatives to develop the workforce should include interprofessional training opportunities[19]. Building from this charge, we designed a locally customised activity that required communication between a general practitioner (doctor) and a community pharmacist to optimise the patient care experience.

This paper describes and evaluates the implementation of this IPE activity across two professions and two countries. The research findings and lessons learnt from our experience respond to a gap in existing IPE literature, which, according to a 2016 review of global health care [7], mostly draws on developed-world undergraduate programs and the nursing profession. Further, whilst there have been numerous publications about IPE from Australia [20–24] and Malaysia [25–29], we identified no published investigations of the overlap of professional and geographical systems with regard to perceptions of collaborative care. Further research in this respect is important as the healthcare workforce in many areas is becoming more culturally diverse due, in part, to migration for improved employment opportunities.

## Materials and methods

### Activity

The design and implementation of the activity were led by a core planning team that included leaders and instructors from both countries to ensure buy-in from the student, instructor, and administrative stakeholders. This team met in person and via Zoom® videoconferencing regularly to monitor the project plan which was accessed via a shared Google® site.

In line with the Monash CCC framework, the learning outcomes of the activity were that by completion, active participants would be able to:

- Analyse similarities and differences between professional roles in the management of respiratory illness
- Propose instances where referral to other professions is indicated
- Communicate with other professions in a respectful, responsive, and responsible manner
- Respectfully seek information from and share information with professional colleagues
- Work with other professions to establish a shared vision for care

To achieve these learning outcomes, the 150-minute activity included four phases in a blended design of the individual, large group, and small group work. The sequential discover/explore/apply/reflect format was adapted from the standard instructional model used in the pharmacy course in both countries.

1. Discover | Pre-class – This comprised a brief preparatory video and readings about asthma management that were complementary to the programmatic standard (single profession) instruction about asthma diagnosis and treatment. It was delivered online via the university’s learning management system. Students completed this on their own time prior to the face-to-face activities. This was designed to take about 30 minutes.
2. Explore | Face-to-face session – This comprised a large group lecture introducing the CCC framework, explaining the healthcare motivations for collaborative care in asthma, and describing the format for the activity. It was delivered face-to-face by one of the activity leads to a mixed-student group, i.e., medicine plus pharmacy. This was designed to be completed in 20 minutes.
3. Apply | Face-to-face session – This comprised a case-based workshop with discussion, hands-on activities, debrief, and deliverables. The patient case used in Australia and Malaysia was similar but included contextualisation to reflect local healthcare practices. Students were allocated to mixed small groups which were facilitated by practitioners from the fields of medicine and pharmacy, at least one of each profession per room. The facilitators had been trained previously in IPE methods. This was designed to be completed in 90 minutes.
4. Reflect | Post-class – This comprised a brief reflection and feedback survey. It was delivered online via the university’s learning management system. Students completed this on their own time after the face-to-face activities. This was designed to be completed in approximately 10 minutes.

The scheduling of the IPE activity was synchronised with the timing of the usual instruction about asthma diagnosis and treatment in both the medicine and pharmacy courses in both countries. We held the face-to-face activities on the same day at the main university campuses in Australia and Malaysia. The country-based activities were designed to run in parallel (i.e. students in Australia were not interacting directly with students in Malaysia). Within countries, there was no explicit expectation of continued student-student communications following the conclusion of the activity.

### Instruments

We measured student perceptions with a pre- and post-activity survey using the validated Student Perceptions of Interprofessional Clinical Education-Revised Instrument, Version 2 (SPICE-R2) instrument provided by Zorek in 2018 based on Zorek *et al.* [30]. We selected this instrument based on relevance to our goals, soundness (i.e., comparative fit, overall reliability, and factor reliability) and ease of use. The instrument consists of 10 items with a 5-point Likert-type response scale allocated to three factors (interprofessional teamwork and team-based practice, roles/responsibilities for collaborative practice, and patient outcomes from collaborative practice)[31]. These factors were inspired by the seminal report of North America’s Interprofessional Education Collaborative[32].

Students responded to the survey via the university learning management system. We collected student and facilitator feedback on the quality of the experience via separate online surveys. This feedback was for quality improvement and is not detailed in this manuscript.

### Participants

All second-year medicine (N = 301 in Australia and N = 107 in Malaysia) and pharmacy students (N = 168 in Australia and N = 117 in Malaysia) participated in the 150-minute educational intervention as part of their required units. Because of an imbalance in the size of the cohorts, in Australia, the mixed groups were seated at tables of six with a 2:1 ratio of medicine to pharmacy students. In Malaysia, the mixed groups were approximately evenly distributed.

Although all students in all cohorts participated in the IPE activity, participation in the research component was elective. Students were provided information about the optional research aspect via the university learning management system and a brief presentation to their class by one of the investigators. Consent was implied by completion of the optional surveys in the university learning management system after reading the participant information and consent form.

### Data analysis

Survey data were managed descriptively and analytically. Descriptive analysis involved the calculation of means, frequencies, and range of scores on the student survey responses. Analytical analysis included a Mann–Whitney U test to assess between-group differences; Wilcoxon Signed Ranks test to determine pre-to-post change in item scores, and a paired samples t-test to detect changes in perception for each of the three factors within the SPICE-R2 tool.

## Results

### Engagement

**Figure 1.**
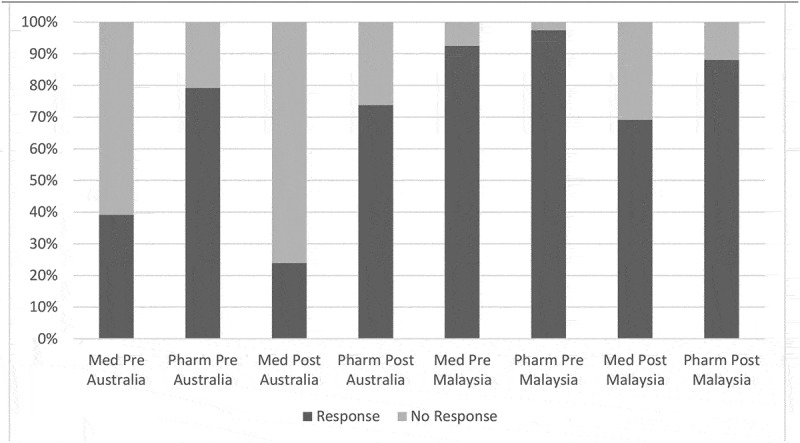
Engagement in the pre- and post-survey by profession and location.

Engagement in the research portion of the activity varied by profession and country of participation (See Figure 1). A total of 326/693 (47%) of students participated in the evaluative research by completing *both* the pre- and post-activity surveys.

### Perceptions

After the activity, we observed positive changes in most items for both medicine and pharmacy students. In particular, there was a significant increase in medicine students’ responses in 8/10 items and overall significantly higher responses from the cohort in Malaysia. Table 1 details the comparative changes in SPICE-R2 responses pre-/post-activity across professions and countries.

**Table 1.** Comparison of changes in student perceptions pre- and post-activity across professions and countries using SPICE-R2.

**Survey item**	**Factor**	**Context**	**Medicine students** Med-AU, n = 43 Med-MA, n = 71			**Pharmacy students** Pharm-AU, n = 109 Pharm-MA, n = 103			
			**Pre** **Med**	**Post** **Med**	**p-value**	**Pre** **Pharm**	**Post** **Pharm**	**p-value**	
1. Working with students from different disciplines enhances my education.	**T**	**Australia**	4.35	4.58	0.043	4.23	4.2	0.987	
		**Malaysia**	4.24	4.49	***0.001***	4.27	4.46	0.013	
2. Patient/client satisfaction is improved when care is delivered by an interprofessional team.	**R**	**Australia**	3.7	4	0.017	3.92	4.06	0.062	
		**Malaysia**	3.89	4.28	***<0.001***	3.9	4.39	***<0.001***	
3. My role within the interprofessional team is clearly defined.	**O**	**Australia**	4.72	4.81	0.206	4.66	4.6	0.621	
		**Malaysia**	4.54	4.59	0.468	4.52	4.56	0.763	
4. Participating in educational experiences with students from different disciplines enhances my ability to work in an interprofessional team.	**T**	**Australia**	4.51	4.51	1	4.39	4.22	0.056	
		**Malaysia**	4.38	4.56	0.029	4.32	4.48	0.042	
5. I have an understanding of the courses taken by and training requirements of other health professionals.	**R**	**Australia**	2.95	3.86	***<0.001***	3.5	4.02	***<0.001***	
		**Malaysia**	3.48	4.27	***<0.001***	3.77	4.33	***<0.001***	
6. Healthcare costs are reduced when patients/clients are treated by an interprofessional team.	**0**	**Australia**	3.09	3.7	***<0.001***	3.48	4.06	***<0.001***	
		**Malaysia**	3.41	3.93	***<0.001***	3.39	3.98	***<0.001***	
7. Health profession students from different disciplines should be educated to establish collaborative relationships with one another	**T**	**Australia**	4.6	4.74	0.058	4.5	4.46	0.86	
		**Malaysia**	4.34	4.55	***0.005***	4.46	4.56	0.194	
8. I understand the roles of other professionals within the interprofessional team.	**R**	**Australia**	3.53	3.81	0.047	3.88	4.13	***0.002***	
		**Malaysia**	3.73	4.35	***<0.001***	3.83	4.46	***<0.001***	
9. Patient/client-centeredness increases when care is delivered by an interprofessional team.	**O**	**Australia**	4.14	4.63	***0.001***	4.33	4.42	0.126	
		**Malaysia**	4.18	4.45	***0.002***	4.34	4.63	***<0.001***	
10. During their education, medical and pharmacy students should be involved in teamwork in order to understand their respective roles.	**T**	**Australia**	4.42	4.72	0.012	4.3	4.39	0.128	
		**Malaysia**	4.21	4.56	***<0.001***	4.37	4.53	0.04	

Factors: T = Interprofessional Teamwork and Team-based Practice, R = Roles/responsibilities for Collaborative Practice, O = Patient Outcomes from Collaborative Practice.

Pre-/Post-activity differences: Wilcoxon Signed Ranks Test (excluding cases list wise) | Median of differences between pre and post: Related Samples Wilcoxon Signed-Rank tests (excluding missing values) | A Bonferroni correction for multiple tests was applied, setting alpha for significance at = <0.005. Statistically significant results are shown in ***bolded italics***.

When we looked more specifically at the three factors described in the SPICE-R2 survey (i.e., factor 1 = teamwork and team-based practice, factor 2 = roles and responsibilities for collaborative practice, and factor 3 = patient outcomes from collaborative practice), after the activity, we observed significant changes in factors 2 and 3 with moderate to large standardised effects (0.44–0.90). Table 2 details these results.

**Table 2.** Comparison of changes in factors pre- and post-activity across countries using SPICE-R2.

#	Factor	Australian campus					Malaysian campus					Both campuses				
		Pre	Post	Change	p-value	d	Pre	Post	Change	p-value	d	Pre	Post	Change	p-value	d
1	Teamwork and team-based Practice	4.39 ± 0.50	4.41 ± 0.67	0.02 ± 0.66	**0.67**	0.03	4.33 ± 0.56	4.52 ± 0.48	0.19 ± 0.54	***<0.001***	0.35	4.36 ± 0.53	4.47 ± 0.58	0.11 ± 0.61	***0.001***	0.19
2	Roles/responsibilities for collaborative practice	3.66 ± 0.61	4.02 ± 0.65	0.35 ± 0.70	***<0.001***	0.51	3.78 ± 0.65	3.78 ± 0.65	0.58 ± 0.64	***<0.001***	0.90	3.72 ± 0.63	4.20 ± 0.60	0.48 ± 0.68	***<0.001***	0.70
3	Patient outcomes from collaborative practice	4.11 ± 0.47	4.37 ± 0.63	0.26 ± 0.59	***<0.001***	0.44	4.07 ± 0.55	4.35 ± 0.51	0.30 ± 0.57	***<0.001***	0.52	4.09 ± 0.51	4.37 ± 0.57	0.28 ± 0.58	***<0.001***	0.48

Paired samples t-test, mean ± SD | Cohen’s d standardised effect size [analysed per Zorek *et al.* [31]]. Statistically significant results are shown in ***bolded italics.***

## Discussion

In implementing and evaluating a large-scale learning activity designed for second-year medical students and second-year pharmacy students based in Australia and Malaysia, we learned several important lessons. First, the activity was logistically challenging but feasible with overall positive outcomes. Working from a shared vision (i.e., starting from the learning objectives of the CCC framework), engaging in careful planning (i.e., coordinating frequent video conference calls with the planning teams across two countries), using familiar platforms (e.g., shared learning management system), and incorporating robust evaluation (i.e., using the validated SPICE-R2 instrument [33]) helped to build community and to justify the value. This is consistent with the intended use of the shared CCC framework [13] and previous guidance[9]. The evaluation data and the quality improvement feedback from students/facilitators have been used to improve this activity for subsequent implementations. It has also been used to design subsequent IPE activities involving other health professions in the university.

Second, the customisation of the activities for different geographies/practice systems/contexts was critical. For example, although instruction on both campuses is in English, the differing healthcare systems in Australia and Malaysia meant that we sometimes used different terminologies. When describing a specific device for delivering inhaled treatments, we called this a ‘puffer’ in Australia and a ‘pump’ in Malaysia to reflect standard practice. More importantly, we had to acknowledge with great care the potential barriers between healthcare professionals in the public/private sectors. For example, since both general practitioner clinics and pharmacies in Malaysia are private businesses, facilitators specifically addressed the potential perception of some competition between professions. The facilitation methods are also likely to have differed somewhat by country, despite using a similar facilitator training process and activity guide. Specifically, the shift from didactic teaching methods to active student learning was reported to be more challenging on the campus in Malaysia. Consistent with the recommendations of Lee *et al.* [34] and Haruta *et al.* [35]. IPE should differ across context. This is not only due to the way different health professions interact in practice settings but also to instructor exposure to other health professions and other instructional methods in the academic setting.

Rather than replicating curriculum across contexts, we acknowledge the need to work towards an equitable, context-sensitive, and locally-driven approach to implementing IPE. [11] For example, we learned through this activity that it was important to show realistic examples of good practice in a culturally appropriate format. One example of a change we introduced in a subsequent implementation of this activity was to show a video example of a potential phone call between a general practice doctor and a community pharmacist to demonstrate a positive Australian interaction. In Malaysia, the team chose to demonstrate a similar message via a live role-play of the scenario.

Third, the variation in improvement across the three factors of the SPICE-R2 instrument may have been influenced by the design of the learning activity. One of the tasks included in the workshop was for the medicine/pharmacy small groups to map out the roles and responsibilities of all the health professionals who were providing care to the patient in the case. This task is directly related to factor 2 (roles/responsibilities for collaborative practice) in which we saw significant improvement. Another workshop task was for the groups to outline the benefits to the patient of the team working together. This task is directly related to factor 3 (patient outcomes from collaborative practice) in which we saw significant improvement. In comparison, factor 1 focuses on teamwork in practice. In Australia, one SPICE-R2 item consistently observed little change, i.e., *‘*participating in educational experiences with students from different disciplines enhances my ability to work on an interprofessional team.’ We suggest that this was related to the fact that our intervention was a brief, one-time, classroom-based activity in students who had not previously worked together and who had, at this point, only limited exposure to direct patient care activities. It also supports the need for interprofessionalism to be integrated across the continuum of health professions courses. Recent study of nursing and pharmacy students working together through multiple stations of high- and medium-fidelity simulation activities suggests that this intensity influenced their respect for collaboration by healthcare team members to improve outcomes[36]. Still, multiple, contextually relevant, experiential work-based learning activities may be necessary to master the hierarchy of knowledge described in the entry-to-practice level of the curriculum framework.

Next, we noted that while engagement in the *activity* was high, participation in the *research* varied by profession and country. This suggests that the way the program was framed by the instructional leaders for each of the four cohorts (i.e., medicine Australia, medicine Malaysia, pharmacy Australia, pharmacy Malaysia) may have differed. One specific difference was that the coordinator of the integrated medical unit in Australia was not directly involved with the design, delivery, or evaluation of the activity. Although he supported conceptually the implementation of the curriculum, his lack of visibility may have influenced student perceptions of the value of contributing to the research. Faculty modelling of the importance of interprofessionalism has been reported to impact on student perceptions in previous studies [37–40].

Finally, our pre-/post-activity results largely align with previous published use of the SPICE instruments [30,31], extending these findings to larger and different cohorts. Differences to note include that we did not find a whole of cohort significant pre-post difference in the item relating to improved patient satisfaction when care is delivered by a team, although the baseline measures in our study were higher. We did find a whole of cohort significant change in two items that prior study did not – i.e., items relating to educating health profession students together to establish a collaborative practice, and involving medical and pharmacy students in teamwork during their education to understand roles. This was the first IPE activity for our students (both campuses) and the carefully constructed addition to the curriculum was positively perceived.

Our study demonstrates an equitable, context-sensitive, and locally driven approach to implementing IPE that other educators may find useful. Still, these results have some limitations. First, we did not collect demographic data from students other than their profession and site. Other demographic factors such as gender, previous study, international status, and ratio of medicine to pharmacy students may also have contributed to our outcomes. In addition, the lower participation rate by medical students in Australia may influence the representativeness of this information. Despite these imperfections, student perceptions about the activity were positive. In particular, participants expressed improved understanding of roles and responsibilities and the potential for better patient outcomes through collaborative care. We capitalised on this when the COVID-19 pandemic of 2020 compelled us to redesign the activity quickly for wholly online delivery[41]. We can also use these results to develop longitudinal activities that bridge from the classroom to health-system settings. Furthermore, we highlight the potential influence of the learning activity design on the variation in improvement across the three factors of the SPICE-R2 instrument and the need to integrate interprofessionalism across the continuum of health professional courses. In so doing, health professions education can better promote and strengthen collaboration for patient care, learning *about, from*, and *with* other members of the healthcare team for better healthcare delivery.
